# Estimated clinical benefit of combining highly conformal target volumes with Volumetric-Modulated Arc Therapy (VMAT) versus conventional flank irradiation in pediatric renal tumors

**DOI:** 10.1016/j.ctro.2021.04.007

**Published:** 2021-05-03

**Authors:** Joeri Mul, Enrica Seravalli, Mirjam E. Bosman, Cornelis P. van de Ven, Annemieke S. Littooij, Martine van Grotel, Marry M. van den Heuvel-Eibrink, Geert O. Janssens

**Affiliations:** a Princess Máxima Center for Pediatric Oncology, Heidelberglaan 25, 3584 CS, Utrecht, the Netherlands; b Department of Radiation Oncology, University Medical Center Utrecht, Heidelberglaan 100, 3584 CX, Utrecht, the Netherlands; c Department of Radiology, University Medical Center Utrecht, Heidelberglaan 100, 3584 CX, Utrecht, the Netherlands

**Keywords:** Pediatric renal tumors, Wilms tumor, VMAT, Conformal radiotherapy, Organs at risk, Side-effects, SIOP-RTSG, International Society of Pediatric Oncology – Renal Tumor Study Group, RT, radiotherapy, AP/PA, Anterior-Posterior/Posterior-Anterior, IMRT, Intensity-Modulated Radiotherapy, VMAT, Volumetric-Modulated Arc Therapy, vs, versus, MRI, Magnetic Resonance Imaging, CT, Computed Tomography, OARs, organs at risk, GTV, Gross Tumor Volume, CTV, Clinical Target Volume, ITV, Internal Target Volume, PTV, Planning Target Volume, TBV, Total Body Volume, PD, Prescribed Dose, ID, integral dose, 95% CI, 95% Confidence Interval

## Abstract

**Background:**

For decades, Anterior-Posterior/Posterior-Anterior (AP/PA) photon beams were standard-of-care for flank irradiation in children with renal cancer. Recently, highly conformal flank target volumes were defined correcting for postoperative organ shift and intra-fraction motion.

By radiotherapy treatment plan comparison, this study aims to estimate the clinical benefits and potential risks of combining highly conformal target volumes with Volumetric-Modulated Arc Therapy (VMAT) versus conventional target volumes with AP/PA beams for flank irradiation.

**Materials and Methods:**

Twenty consecutive renal tumor cases (left/right-sided:10/10; median age:3.2 years) were selected. Highly conformal flank target volumes were generated for VMAT, while conventional target volumes were used for AP/PA. For each case, the dose to the organs at risk (OARs) and Total Body Volume (TBV) was calculated to compare VMAT with AP/PA treatment plans for a prescribed dose (PD) of 14.4/1.8 Gy. Dose constraint violation of the tail of the pancreas and spleen (D_mean_ < 10 Gy), heart (D_50_ < 5 Gy) or mammary buds (D_mean_ < 10 Gy) were prioritized as potentially beneficial for clinics.

**Results:**

Highly conformal Planning Target Volumes (PTV) were smaller than conventional volumes (mean ΔPTV_AP/PA_-PTV_VMAT_: 555 mL, Δ60%, p=<0.01). A mean dose reduction favoring VMAT was observed for almost all OARs. Dose constraints to the tail of the pancreas, spleen, heart and mammary buds were fulfilled in 8/20, 12/20, 16/20 and 19/20 cases with AP/PA, versus 14/20, 17/20, 20/20 and 20/20 cases with VMAT, respectively. In 12/20 cases, VMAT prevented the dose constraint violation of one or more OARs otherwise exceeded by AP/PA. VMAT increased the TBV receiving 10% of the PD, but reduced the amount of irradiated TBV for all higher doses.

**Conclusion:**

Compared to 14.4 Gy flank irradiation using conventional AP/PA photon beams, an estimated clinical benefit by dose reduction to the OARs can be expected in 60% of the pediatric renal tumor cases using highly conformal flank target volumes combined with VMAT.

## Highlights

• Recently, flank target volumes adjusted for organ shift/motion have been defined.
• Highly conformal volumes with VMAT were compared to conventional volumes/beams.
• The new approach prevented a dose constraint violation of ≥ 1 OARs in 60% of cases.
• VMAT reduced the irradiated Total Body Volume receiving > 10% of the prescribed dose.

## Introduction

Renal cancer is diagnosed in five percent of children presenting with cancer [1]. Patients treated according to protocols of the International Society of Pediatric Oncology Renal Tumor Group (SIOP-RTSG) receive 4 to 6 weeks of preoperative chemotherapy followed by a nephrectomy with lymph node sampling. Depending on tumor stage and histology, adjuvant chemotherapy is administered with or without radiotherapy (RT) [2]. After successive studies, increased awareness of side effects and better treatment outcomes have led to a safe reduction of treatment intensity for most renal tumor types [2], [3], [4], [5], [6], [7]. As of now, flank irradiation is administered to 20–25% of patients with pediatric renal tumors with cumulative fractionated doses between 10.8 Gy and 25.2 Gy [2], [8].

For decades, conventional two-opposing Anterior-Posterior/Posterior-Anterior (AP/PA) photon beams have been standard-of-care to cover the flank target volume [9]. Based on current SIOP-protocols, flank target volumes are generated from the projection of the primary tumor after preoperative chemotherapy on a two- or three-dimensional plane without adapting for postoperative changes. However, renal tumors arise from the retroperitoneal area and rarely invade the intraperitoneal structures. After preoperative chemotherapy, the kidney is removed with a very limited risk of intraoperative rupture and surrounding organs shift into the operative bed [10]. Consequently, most of the irradiated volume in case of an AP/PA approach and conventional target volumes consists of healthy tissue.

**Fig. 1:**
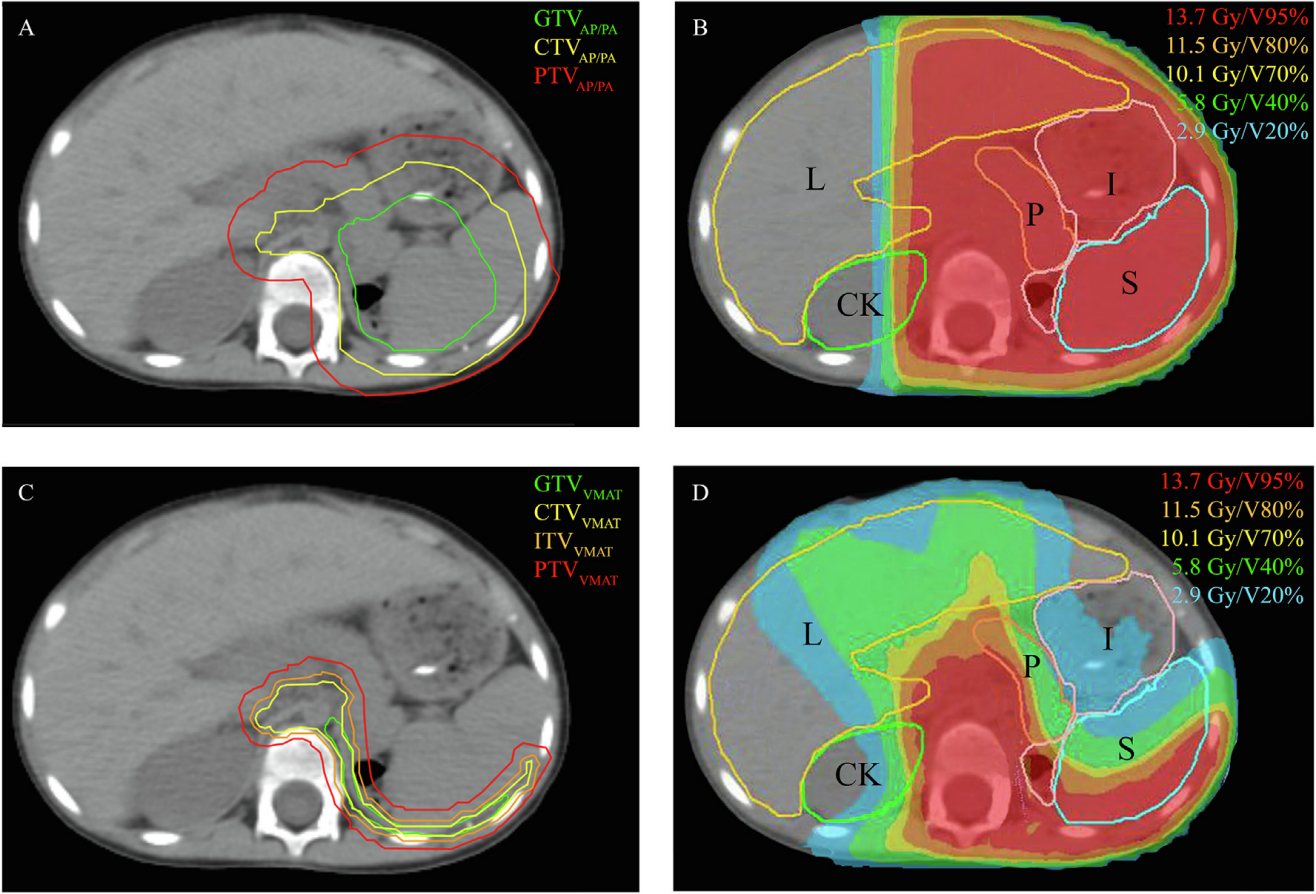
All diagrams show the axial CT-scan after surgery of one patient with a renal tumor originating from the left kidney. Conventionally, target volumes based on the conventional delineation approach (1A) are irradiated using an AP/PA photon beam dose distribution (1B). With the highly conformal method, target volumes are corrected for postoperative shift of organs (1C) and irradiated using a VMAT dose distribution (1D). Isodoses are shown as 95% (13.7 Gy, red), 80% (11.5 Gy, orange), 70% (10.1 Gy, yellow), 40% (5.8 Gy, green) and 20% (2.9 Gy, blue) of the prescribed dose. Abbreviations: AP/PA, Anterior-Posterior/Posterior-Anterior; VMAT, Volumetric-Modulated Arc Therapy; Gy, Gray; GTV, Gross Tumor Volume (green line); CTV, Clinical Target Volume (yellow line); ITV, Internal Target Volume (orange line); PTV, Planning Target Volume (red line); L, liver; I, intestines; CK, contralateral kidney; S, spleen. (For interpretation of the references to colour in this figure legend, the reader is referred to the web version of this article.)

The increasing availably of rotational Intensity-Modulated Radiotherapy techniques like Volumetric-Modulated Arc Therapy (VMAT), and improved diagnostics like Magnetic Resonance Imaging (MRI) and 4D-Computed Tomography (CT) have resulted in the development of a highly conformal target volume definition for flank irradiation [8]. Combined with modern RT techniques, these new target volumes might reduce the dose and potentially associated late toxicity to healthy tissue (Fig. 1). While a single center analysis provides encouraging evidence that an excellent locoregional control, equal to the SIOP-2001 and AREN0532 trials, can be obtained by this approach, an estimation of the clinical benefit of this approach is lacking [3], [11], [12].

The combination of conventional versus (vs.) highly conformal target volumes and AP/PA vs. VMAT techniques may result in four different scenarios. Given the limited benefit of OAR sparing by the use of highly conformal target volumes combined with an AP/PA technique or VMAT without highly conformal target volumes with adjustment for the organs at risk, this study aims to estimate the clinical benefit and risk of combining highly conformal flank target volumes with VMAT vs. conventional target volumes with AP/PA beams.

## Materials and methods

### Patient selection

Twenty consecutively selected patients with unilateral renal tumors who had received preoperative chemotherapy, nephrectomy with lymph node sampling followed by postoperative chemotherapy and flank irradiation at the University Medical Centre Utrecht and Princess Maxima Centre for Pediatric Oncology (Utrecht, The Netherlands) between April 2015 and November 2017 were included in this analysis (institutional review board approval number: WAG/mb/17/008865). In all patients, surgical clips were placed at the lateral and superior border of the operative bed to optimize highly conformal target volume delineation [2], [8].

### Image characteristics

For each patient, a T1-weighted MRI scan (Achieva 1.5 T, Philips Medical Systems, Best, The Netherlands, slice thickness of 1.5 mm) with and without the administration of gadolinium contrast agent was acquired after chemotherapy and before surgery. This MRI scan was rigidly co-registered to a postoperative planning CT-scan in supine RT treatment position (Brilliance, Philips Medical Systems, Best, The Netherlands, slice thickness of 2–3 mm). Respiratory trace measurements for pulmonary gating were obtained using a deformable rubber belt fixed to the patient's chest to allow 4D-CT imaging (Philips Bellow System, Best, The Netherlands). Each complete respiratory cycle during spontaneous breathing was captured as a series of ten equally distributed time intervals. The planning-CT was generated by taking the pixel-by-pixel average of all ten phases of the 4D-CT. Both planning-CT and 4D-CT-scans shared the same spatial coordinates, therefore, an additional co-registration step was not necessary. For treatment positioning, daily pre-treatment CBCT-scans were acquired with an arc of 200 degrees of 10 ms using 16 mA with 100 kV and an acquisition timeframe of 30 s for all treatment fractions using the Elekta XVI 4.5.1 on-board CBCT imaging system (Elekta, Stockholm, Sweden). All patients were immobilized in supine position by a vacuum mattress during treatment.

### Delineation of target volumes, organs at risk (OARs) and Total body volume (TBV)

Information on lymph node involvement and resection margins was gathered from the surgery and pathology reports to determine target volume extension. For each patient, two different approaches for target volume delineation were used. For the AP/PA plan, a gross tumor volume (GTV_AP/PA_) and a clinical target volume (CTV_AP/PA_) were delineated based on the SIOP-RTSG UMBRELLA-2016 protocol recommendations and expanded with a Planning Target Volume (PTV_AP/PA_) margin of 10 mm [2], [13]. For the VMAT plan, a GTV_VMAT_, a CTV_VMAT_ and an Internal Target Volume (ITV_VMAT_) were generated according to the highly conformal flank target volume definitions, as recently published [8]. The PTV_VMAT_ was defined as a 5 mm expansion from ITV_VMAT_. The method of target volume delineation for both treatment modalities is described in Supplementary Table 1.

The contralateral kidney, intestines, pancreas (head and tail), spleen, liver, heart and both mammary buds were delineated as OARs. The medial border of the pancreatic tail was defined by a dorsoventral tangential to the left side of the vertebral body [14]. Intestines were delineated from the cranial to the caudal margin of the PTV_AP/PA_, while the vertebrae were separately delineated for each treatment plan from the cranial to caudal margin of the corresponding PTV. A total body volume (TBV) was defined as the external body contour 10 cm above and below the PTV_AP/PA_. For the delineating target volumes and OARs, CT gray-level mapping was standardized to a window/level of 250/40 Hounsfield units, respectively.

### Treatment planning

Treatment plans were generated using the Monaco treatment planning system (Elekta Instrument AB Stockholm, Sweden, version 5.11.02). For *in silico* comparison of both techniques, a prescribed dose (PD) of 14.4 Gy in 1.8 Gy fractions was used for all cases, since it is indicated in the majority of patients that require flank irradiation [2], [3]. No additional boost doses were planned in case of any residual macroscopic tumor. Conventional AP/PA plans consisted of two-opposing 10 MV photon fields in anterior and posterior direction. VMAT plans consisted of a full-arc 10 MV photon arc and were optimized for the dose constraints depicted in Table 1. Target volume coverage was considered adequate if 95% of the PD was given to at least 99% and 95% of the CTV (V95%≥99%) and PTV (V95%≥95%), respectively. To avoid asymmetric growth, a homogenous dose corresponding to 95% of the PD was used to enclose the vertebrae in the left–right and ventrodorsal dimensions for both techniques .

**Table 1:** Dosimetric criteria applied for the OARs and their associated increased risk of late effects in childhood cancer survivors.

OARs [reference]	Constraints	Late effects	Reported risk increase [95% CI]	Cumulative incidence
Contralateral kidney [13], [16]	D_mean_ < 12 Gy	Renal function impairment	Unknown	Unknown
Intestines [17]	D_mean_ < 20 Gy	Intestinal occlusion requiring surgery	20.0–29.9 Gy vs. 0 Gy: RR 2.2 [1.2, 4.3]	20.0–29.9 Gy: 5.8% at 35 years from diagnosis
Pancreas (tail) [18], [19]	D_mean_ < 10 Gy	Diabetes mellitus	10.0–19.9 Gy vs. 0 Gy: RR 6.8 [2.3, 19.9]	10.0–19.9 Gy: 12.7% at 45 years of age
Spleen [20], [21]	D_mean_ < 10 Gy	Late infection-related mortality	10.0–19.9 Gy vs. 0 Gy: RR 5.5 [1.9, 15.4]	10.0–19.9 Gy: 1.1% at 35 years of age
Liver [13], [22]	D_mean_ < 20 Gy	Hepatotoxicity	Unknown	Unknown
Heart [15], [23]	D_mean_ < 10 Gy*or*D_50_ < 5 Gy	Any cardiac disease	10.0–19.9 Gy vs. 0 Gy: RR 2.2 [1.6, 2.9]*or*D_50_ < 5 Gy vs. D_50_ > 5 Gy: RR 1.6 [1.1, 2.3]	10.0–19.9 Gy: 5.8% at 30 years*or*D_50_ > 5 Gy: 4.0% at 30 years
Mammary buds [24], [25]	D_mean_ < 10 Gy	Invasive breast cancer	10.0–19.9 Gy vs. 0 Gy: OR 6.5 [2.3, 18.5]	Unknown

**Abbreviations:** OARs, organs at risk; 95% CI, 95% Confidence Interval; D_mean_, mean dose; Gy, Gray.

### Plan evaluation

For each case, dose-volume-histograms were calculated for both AP/PA and VMAT treatment plans on the planning CT. For all OARs, the mean dose was computed, while for the heart the dose received by 50% of the volume (D50) was used in line with Bates et al. [15]. The TBV receiving 100% to 10% of the PD (i.e. V100% to V10%, respectively) was calculated using 10% decremental steps, as well as the integral dose of the TBV (TBV_ID_). The TBV_ID_ was defined as:

$$\mathrm{TB}V_{\mathrm{ID}}\;\left[\mathrm{Gy}*L\right]=D_{\mathrm{mean}}TBV\rho$$

where ρ is the body density which was assumed to be uniform (1 g/cm^3^).

For subgroup comparison, individual patient data was collected on tumor location (left- vs. right-sided) or lymph node involvement (LN + vs. LN-). Fulfillment of the dose constraints of one or more OARs in favor of one technique was considered to be of potential clinical relevance based on the evidence summarized in Table 1 [13], [15], [16], [17], [18], [19], [20], [21], [22], [23], [24], [25].

### Statistical analysis

The size of highly conformal and conventional target volumes (in mL), as well as the mean dose to the OARs and TBV (in Gy) between VMAT and AP/PA treatment plans, were compared. For normally distributed data, paired samples T-test was used, while the Wilcoxon Signed-Ranks Test was used in case of non-normal distributed data. A two-tailed p-value of < 0.05 indicated statistical significance. Data were analyzed using statistical software SPSS version 25.0 for Windows (SPSS, INC, Chicago, IL, USA).

## Results

### Patient and tumor characteristics

Target volumes and two treatments plans were generated using the planning CT’s of twenty consecutive cases with renal tumors (median age: 3.2 years; male/female: 12/8; left/right-sided: 10/10; LN+/LN-: 15/5).

### Target volume comparison

Highly conformal target volumes intended for VMAT were smaller compared to target volumes used for the AP/PA (mean GTV_VMAT_ vs. GTV_AP/PA_: 52 mL vs. 261 mL, p = 0.04; mean CTV_VMAT_ vs. CTV_AP/PA_: 142 mL vs. 488 mL, p = <0.01; mean PTV_VMAT_ vs. PTV_AP/PA_: 376 mL vs. 931 mL, p = <0.01) (Table 2).

**Table 2:** Target volume comparison.

Target volumes	VMAT (in mL)	AP/PA (in mL)	p-value
GTV*			
mean	52	261	0.04
min–max	8–245	24–1149	

CTV			
mean	142	488	<0.01
min–max	34–681	138–1717	

PTV			
mean	376	931	<0.01
min–max	115–1529	320–2898	

*The GTV_AP/PA_ are based on the preoperative tumor dimensions (GTV_pre_), whereas the GTV_VMAT_ also accounts for postoperative changes (GTV_post_).

Abbreviations: VMAT, Volumetric-Modulated Arc Therapy; AP/PA, Anterior-Posterior/Posterior-Anterior; mL, milliliter; GTV, gross tumor volume; CTV, clinical target volume; PTV, planning target volume.

### Target Coverage, dose to the OARs and TBV

The CTV and PTV coverage by the 95% isodose was adequate for all patients and both treatment planning techniques (VMAT: mean CTV_V95%_: 99.9%, range: 98.4% − 100.0%, mean PTV_V95%_: 99.5%, range: 98.6% − 99.9% vs. AP/PA: CTV_V95%_: 99.9%, range: 98.6% − 100.0%; PTV_V95%_: 97.9%, range: 95.1% − 99.3%).

For the whole group of 20 cases, a mean dose reduction in favor of VMAT was observed for the contralateral kidney (ΔAP/PA-VMAT: 1.7 Gy, p = <0.01), intestines (ΔAP/PA-VMAT: 3.4 Gy, p = <0.01), tail of the pancreas (ΔAP/PA-VMAT: 2.4 Gy, p = <0.01), spleen (ΔAP/PA-VMAT: 1.7 Gy, p = 0.03) and heart (ΔAP/PA-VMAT: 2.4 Gy, p = <0.01) (Supplementary Table 2).

Compared to AP/PA, VMAT was more frequently able to fulfill the constraints to the tail to the pancreas (AP/PA vs. VMAT: 8/20 vs 14/20), the spleen (AP/PA vs. VMAT: 12/20 vs 17/20), the heart (AP/PA vs. VMAT: 16/20 vs. 20/20) and mammary buds (AP/PA vs VMAT: 19/20 vs 20/20). In 12/20 cases, VMAT demonstrated a potential clinical benefit by fulfilling the dose constraint of one or more OARs otherwise violated by AP/PA (Table 3).

**Table 3:** Tumor characteristics and dose to the OARs; per case and per technique.

Cases				Dose to the OARs (in Gy)								
#	Laterality	LN	Technique	Contralateral kidney	Intestines	Tail of pancreas	Spleen	Liver	Heart	Heart	Mammary bud, left	Mammary bud, right
				*mean*	*mean*	*mean*	*mean*	*mean*	*mean*	*D50*	*mean*	*mean*
**1**	Left	LN+	*VMAT*	5.2	6.6	**9.9**	**4.4**	4.1	0.2	0.2	n.a.	n.a.
			*AP/PA*	7.9	13.3	14.6*	**14.7**†	6.0	6.0	3.6		
**2**	Left	LN+	*VMAT*	4.4	9.2	10.4*	13.0†	5.7	**0.5**	**0.4**	n.a.	n.a.
			*AP/PA*	7.2	13.0	14.4*	14.7†	6.7	**11.0**‡	**13.5**‡		
**3**	Left	LN+	*VMAT*	3.8	8.1	**9.3**	11.2†	4.8	0.5	0.4	n.a.	n.a.
			*AP/PA*	7.9	12.0	14.4*	13.7†	3.9	0.7	0.5		
**4**	Left	LN+	*VMAT*	3.0	8.4	10.9*	**9.9**	4.7	0.6	0.3	n.a.	n.a.
			*AP/PA*	3.3	12.2	14.4*	**14.5†**	5.2	3.5	1.1		
**9**	Left	LN-	*VMAT*	3.1	7.8	10.5*	**8.6**	2.9	0.2	0.2	n.a.	n.a.
			*AP/PA*	11.7	14.0	14.9*	**14.7†**	5.5	3.3	1.1		
**13**	Left	LN+	*VMAT*	4.5	7.6	**9.9**	5.4	4.9	0.4	0.2	0.2	0.3
			*AP/PA*	5.5	11.8	14.6*	7.1	5.3	1.1	0.5	1.2	0.8
**14**	Left	LN+	*VMAT*	2.5	9.3	12.8*	12.2†	4.8	0.5	0.3	0.1	0.1
			*AP/PA*	1.3	11.9	14.5*	14.1†	4.3	1.9	0.7	0.8	0.2
**16**	Left	LN+	*VMAT*	5.2	7.0	13.6*	**7.9**	7.7	1.5	**0.9**	n.a.	n.a.
			*AP/PA*	11.0	12.2	14.7*	**12.8†**	10.3	7.5	**7.0**‡		
**17**	Left	LN+	*VMAT*	4.1	6.9	**9.1**	3.8	4.6	0.4	0.3	0.5	0.1
			*AP/PA*	5.6	11.3	14.4*	6.7	3.8	1.2	0.5	0.9	0.7
**19**	Left	LN+	*VMAT*	2.8	9.2	**7.5**	**8.0**	5.0	0.5	0.4	n.a.	n.a.
			*AP/PA*	3.7	12.2	14.2*	**12.9†**	4.8	2.2	0.8		
**5**	Right	LN+	*VMAT*	5.2	7.4	10.1*	1.8	6.3	0.3	0.3	0.2	0.2
			*AP/PA*	8.5	11.3	13.3*	0.9	10.1	1.0	0.5	1.0	1.0
**6**	Right	LN-	*VMAT*	2.4	4.1	5.2	1.3	8.8	1.4	**0.9**	0.5	**1.0**
			*AP/PA*	2.1	3.7	3.7	0.7	13.6	7.2	**7.2**‡	0.9	**12.0**§
**7**	Right	LN-	*VMAT*	1.7	6.9	3.1	0.6	7.4	0.1	0.2	0.1	0.2
			*AP/PA*	2.8	8.8	3.5	0.5	8.9	0.3	0.3	0.3	1.2
**8**	Right	LN+	*VMAT*	4.5	5.9	4.9	0.7	4.1	0.1	0.1	n.a.	n.a.
			*AP/PA*	5.1	8.6	6.6	0.5	8.8	0.2	0.3		
**10**	Right	LN+	*VMAT*	3.9	7.7	6.5	1.0	3.9	0.1	0.2	n.a.	n.a.
			*AP/PA*	6.5	9.5	6.3	0.8	8.3	0.3	0.3		
**11**	Right	LN-	*VMAT*	2.4	6.4	1.1	0.8	6.6	0.1	0.1	n.a.	n.a.
			*AP/PA*	2.4	9.6	2.3	0.4	11.8	0.4	0.3		
**12**	Right	LN+	*VMAT*	3.2	5.0	1.9	1.5	6.6	4.5	**2.5**	1.0	3.3
			*AP/PA*	1.0	7.2	1.9	0.5	9.3	7.2	**6.6**‡	0.9	1.7
**15**	Right	LN+	*VMAT*	3.9	7.0	4.3	3.7	8.6	1.6	0.7	n.a.	n.a.
			*AP/PA*	3.3	9.8	4.9	0.9	13.0	6.0	4.0		
**18**	Right	LN-	*VMAT*	2.6	6.5	**9.7**	0.9	4.1	0.1	0.1	n.a.	n.a.
			*AP/PA*	3.0	9.0	11.0*	0.5	8.3	0.2	0.1		
**20**	Right	LN+	*VMAT*	4.3	7.9	5.5	1.8	7.2	0.4	0.3	0.3	0.2
			*AP/PA*	5.9	10.0	4.6	0.9	9.6	1.1	0.5	0.9	1.0

All clinically relevant differences between VMAT and AP/PA are in bold.

* Indicates that the mean dose to the tail of the pancreas is ≥ 10.0 Gy.

† Indicates that the mean dose to the spleen is ≥ 10.0 Gy.

‡ Indicates that the mean dose of the heart is ≥ 10.0 Gy or the D50 is ≥ 5.0 Gy.

§ Indicates that the mean dose to the mammary gland is ≥ 10.0 Gy.

Abbreviations: Gy, Gray; OARs, organs at risk; LN, lymph node involvement; VMAT, Volumetric-Modulated Arc Therapy; AP/PA, Anterior-Posterior/Posterior-Anterior photon beam radiotherapy; n.a., not applicable.

**Fig. 2:**
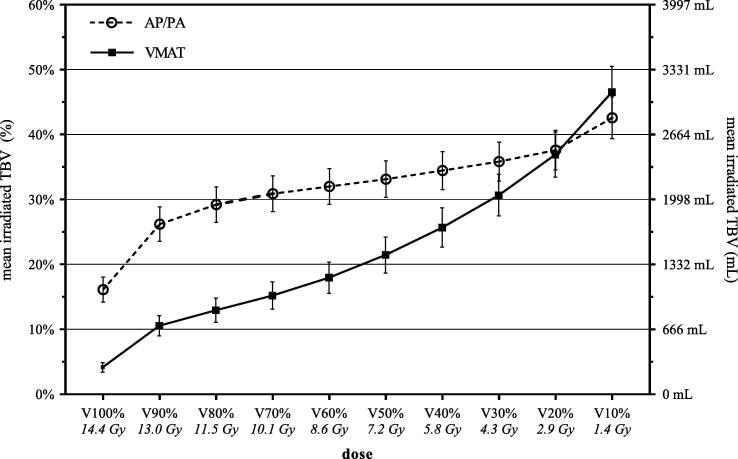
The amount of total body volume in percentage (left y-axis) and milliliter (right y-axis) receiving a specific radiotherapy dose (x-axis, relative percentage of the prescribed dose with absolute dose is shown) (n = 20). Symbols depict the group mean irradiated TBV for the AP/PA treatment plans (circles) and the VMAT treatment plans (squares). Error bars represent 95% confidence interval. Abbreviations: mL, milliliter; VMAT, Volumetric-Modulated Arc Therapy; AP/PA, Anterior-Posterior/Posterior-Anterior photon beam radiotherapy.

Fig. 2 illustrates that highly conformal target volumes irradiated with VMAT increased the mean TBV receiving up to 10% of the PD compared to conventional target volumes combined with AP/PA. In contrast, mean TBV receiving doses above 20% of the PD were always in favor of VMAT. The median TBV_ID_ was higher for AP/PA (5.1 Gy * L) compared to VMAT (3.6 Gy * L, p = <0.01).

## Discussion

Highly conformal delineation, taking the postoperative shift of organs and intra-fraction motion into account, results in a significant target volume reduction compared to a conventional approach. When combined with VMAT, these new target volumes also reduce the dose to the surrounding organs and the Total Body Volume compared to the conventional approach. Even for a prescribed dose as low as 14.4 Gy, an estimated clinical benefit can be obtained with VMAT in 60% of the cases by fulfilling the dose constraint of at least one OARs otherwise violated by AP/PA.

Although indications for flank irradiation have been refined and doses reduced since SIOP-1 (1971–1974), RT by AP/PA photon beams has remained the gold standard up to now [9]. Due to the limited conformity of this technique, target volumes used for conventional flank irradiation have not been adapted to reflect the postoperative situation [13]. Nevertheless, accurate CTV delineation excluding uninvolved organs is of utmost importance to capitalize on the favorable dose distributions of modern RT techniques. For flank irradiation, the current study demonstrates that the new flank target volume definition allowed a 60% mean decrease in PTV compared to the PTV based on the conventional flank target volume definition [8], [13]. Over the last years, similar efforts have been done to translate craniospinal axis target volumes and Hodgkin lymphoma from the 2/3D period into the era of high conformality [26], [27], [28].

Historically, two-thirds of the pediatric renal tumor survivors who received abdominal RT developed late adverse effects [6], [7], [29], [30], [31], [32]. Musculoskeletal defects like scoliosis and tissue hypoplasia are among the most common late effects observed [6], [33]. Since the PTVs overlap with the vertebrae and ribs and dose gradients on the primary ossifications centers of the vertebrae should be restricted to 3 or 5 Gy, it is not expected that modern techniques will alter the risk of musculoskeletal problems [34].

Waas et al. demonstrated that Wilms tumor and neuroblastoma survivors who had received abdominal RT more frequently developed components of the metabolic syndrome (i.e. adiposity, hypertension, dyslipidemia or insulin resistance/type 2 diabetes) compared to unirradiated survivors, even if only a part of the pancreas had been irradiated [35]. These components of metabolic syndrome are known risk factors of cardiovascular disease [36], [37], [38], [39]. De Vathaire et al. showed that the cumulative incidence of diabetes mellitus in survivors by the age of 45 that had received between 10 and 19.9 Gy to the tail of the pancreas was 12.7% compared to 1.3% in the unirradiated group [18]. In the current study, 50% less cases acquired a mean dose to the tail of the pancreas above 10 Gy using VMAT instead of AP/PA. Moreover, in the study of Bates et al., the risk of coronary artery disease and heart failure was increased by 60% if>50% of the heart volume received a mean radiation dose above 5 Gy [15]. In our study, this heart constraint was not exceeded in any case using VMAT versus 20% of cases with the AP/PA approach. This implies that VMAT might potentially reduce the incidence of cardiovascular disease in survivors of pediatric renal cancer, by respecting dose constraints to the tail of the pancreas and the heart.

Furthermore, Weil et al. demonstrated that childhood cancer survivors who had received a mean dose of 10–19.9 Gy to the spleen had a 5.5 times higher risk of infection-related late mortality than the unirradiated survivors [20]. In the current study, 80% of the left-sided cases using AP/PA had a spleen dose ≥ 10 Gy compared to 30% using VMAT. Therefore, VMAT might lower the risk of functional asplenia and, subsequently, may restrict the need for immunization or prophylactic antibiotics in patients with a left-sided renal tumor [21].

This radiotherapy treatment plan comparison highlights that, even though the mean dose reduction to the OARs for the whole group was limited to 3.0 Gy, the estimated benefit for individual patients can be clinically relevant. Current data on late toxicity often originates from patient cohorts treated with higher RT doses, meaning that the dose–response relationship for lower doses has not been fully understood for most OARs. As a result, the clinical benefit of 3, 5 or more Grays by a rotational IMRT technique for flank irradiation using a PD of 14.4 Gy remains unclear for the kidneys, intestines and liver based on the current evidence available. More benefit of rotational IMRT techniques is expected for children with high-risk renal tumors who receive a PD of 25.2 Gy, since the dose constraints for the contralateral kidney, intestines and liver will also become relevant [2].

While this study has shown the potential benefits of VMAT and highly conformal target volumes, some disadvantages of this approach may exist. Firstly, a recent multicenter international exercise on highly conformal flank target volume delineation showed that the variability among clinicians is a matter of concern and results in an underestimation of the area at risk in more than half of the delineations [40]. To assess the impact of inter-clinician variability on locoregional control in a multicenter setting, the SIOP-RTSG will organize a prospective observational study using centralized review of the highly conformal target volumes and dose distribution before onset of radiotherapy. Secondly, although the use of VMAT vs. AP/PA hardly makes any difference in irradiation time, it is true that daily online imaging easily adds ± 5 min compared to daily positioning using surface markers and laser lines only. This may increase the risk of intrafraction movement and missing of the target area. For this reason, children are immobilized in a supine position using a vacuum mattress and only children with proven compliance will be treated without anesthesia. By this approach, it was previously demonstrated by pre- and post-treatment imaging that intrafraction uncertainties are reduced to a minimum [41], [42]. Thirdly, at this time, it could be argued that the benefit of the new approach for flank irradiation may be disproportional to the time and effort for radiation oncologists to generate and execute highly conformal treatment plans. In the near future, it is expected that treatment preparation time will be reduced with the introduction of artificial intelligence methods enabling auto-contouring of all abdominal organs at risk and ultimately standard flank target volumes as well [43]. Finally, it is hypothesized that rotational IMRT techniques like VMAT may increase the risk of a subsequent malignant neoplasm (SMN) compared to conventional RT techniques due to an increase in low dose irradiated volume [44], [45], [46], [47]. Indeed, in the current analysis, the TBV receiving up to 10% of the PD (i.e. ~ 2 Gy) was increased by VMAT. However, for higher doses, a VMAT dose distribution becomes increasingly superior to an AP/PA dose distribution, leading to a significantly lower integral dose in the TBV and even reduces the TBV receiving 90% of the PD by>50%. Since SMN are mainly observed in intermediate to high dose RT areas, the benefit from the reduction of high dose irradiation could even outweigh the slight increase of low dose to a larger volume [31], [48], [49], [50]. Although the dose–response relationship for induction of secondary cancers has been widely debated, there are no validated models yet available to predict the absolute reduction of risk [48]. Recent studies have shown that a further dose reduction to the abdominal organs can also be obtained by the use of proton therapy [51], [52]. Nevertheless, respecting time to onset of radiotherapy per protocol, but also technical issues like diaphragmatic motion and tissue density changes, remain challenges for referral for proton therapy on a routine base.

In conclusion, this radiotherapy treatment plan comparison demonstrates that, for a prescribed dose of 14.4 Gy, Volumetric-Modulated Arc Therapy with target volumes adapted to the postoperative situation can achieve a potential clinical benefit over conventional target volumes with Anterior-Posterior/Posterior-Anterior photon beams in 60% of the cases by preventing dose constraint violation of the pancreas, spleen, heart or the mammary buds. Implementing highly conformal flank radiotherapy techniques in clinic demands a prospective follow-up with focus on loco-regional control and registration of radiotherapy-related morbidity.

## Declaration of Competing Interest

The authors declare that they have no known competing financial interests or personal relationships that could have appeared to influence the work reported in this paper.
