# Supplementary material for: Estimated clinical benefit of combining highly conformal target volumes with Volumetric-Modulated Arc Therapy (VMAT) versus conventional flank irradiation in pediatric renal tumors
Source: Clin Transl Radiat Oncol. 2021 May 3;29:20–6. doi: 10.1016/j.ctro.2021.04.007 (PMC8134033; doi:10.1016/j.ctro.2021.04.007)
Supplement: Supplementary data 1 [file mmc1.docx]

# Supplementary table 1

Overview of the conventional and highly conformal flank target volume definitions.

| **Target volume** | **Subsection** | **Conventional delineation for AP/PA radiotherapy** | **Highly conformal delineation for VMAT** |
| --- | --- | --- | --- |
| **GTV** | **Preoperative (GTV_pre_)** | Preoperative macroscopic disease after chemotherapy. | Preoperative macroscopic disease after chemotherapy. |
|  | **Postoperative (GTV_post_)** | Not applicable | Contact zone of GTV_pre_ with all uninvolved OARs removed. |
|  |  |  | - Lateral margin: is defined by lateral clip. |
|  |  |  | - Check surgical report for adhesions or infiltration: include contact zone of the GTV_pre_ with the involved organ. |
| **CTV** | **Tumor (T)** | GTV-T_pre_ + 10 mm. | GTV-T_post_ +10 mm with removal of all uninvolved OARs. |
|  |  | - No CTV expansion inside vertebrae or outside the body. | - Posterior wall: GTV-T_post_ +5 mm (adhesions) or +10 mm (invasion). |
|  |  |  | - Healthy-appearing kidney: GTV-T_post_ +20 mm. |
|  |  |  | - Involved OARs: GTV-T_post_ + 5 mm (adhesions) or +10 mm (invasion). |
|  | **Lymph node area (N)** | GTV-N_pre_ + 10 mm. | GTV-N_post_ +10 mm with removal of all uninvolved OAR. |
|  |  | - Lymph node area around abdominal aorta, inferior vena cava and ipsilateral renal vessels up to T10/11 and down to aorta bifurcation. | - Lymph node area around abdominal aorta, inferior vena cava and ipsilateral renal vessels up to T10/11 and down to aorta bifurcation. |
| **ITV** |  | Not applicable | With surgical (superior) clip and 4D-CT technology: individual margins. |
| **PTV*** |  | CTV + 10 mm. | ITV + 5 mm. |
| *PTV margins may differ per institute depending on the techniques used to correct for uncertainties. The CTV to PTV margins depicted in the table above were used for the current study.  Abbreviations: AP/PA, Anterior-Posterior/Posterior-Anterior; VMAT, Volumetric Modulated Arc Therapy; GTV_pre/post_, Gross Tumor Volume of the primary tumor (T) or lymph node area (N) before and after surgery, respectively; CTV, Clinical Target Volume; ITV, Internal Target Volume; PTV, Planning Target Volume; OARs, Organs At Risk; 4D-CT, four-dimensional Computerized Tomography. | | | |

# Supplementary table 2

Mean dose to the OARs.

| **OARs** | **VMAT (in Gy)** | **AP/PA (in Gy)** | **p-value** |
| --- | --- | --- | --- |
| Contralateral kidney |  |  |  |
| D_mean_ [95% CI] | 3.6 [3.1, 4.1] | 5.3 [3.9, 6.7] | <0.01 |
| min-max | 1.7 – 5.2 | 1.0 – 11.7 |  |
| Intestines |  |  |  |
| D_mean_ [95% CI] | 7.2 [6.6, 7.9] | 10.6 [9.4, 11.7] | <0.01 |
| min-max | 4.1 – 9.3 | 3.7 – 14.0 |  |
| Tail of the pancreas |  |  |  |
| D_mean_ [95% CI] | 7.8 [6.2, 9.5] | 10.2 [7.8, 12.6] | <0.01 |
| min-max | 1.1 – 13.6 | 1.9 – 14.9 |  |
| Spleen |  |  |  |
| D_mean_ [95% CI] | 4.9 [2.9, 6.9] | 6.6 [3.6, 9.7] | 0.03 |
| min-max | 0.6 – 13.0 | 0.4 – 14.7 |  |
| Liver |  |  |  |
| D_mean_ [95% CI] | 5.6 [4.9, 6.4] | 7.9 [6.5, 9.3] | <0.01 |
| min-max | 2.9 – 8.8 | 3.8 – 13.6 |  |
| Heart |  |  |  |
| D_mean_ [95% CI] | 0.7 [0.2, 1.2] | 3.1 [1.6, 4.6] | <0.01 |
| min-max | 0.1 – 4.5 | 0.2 – 11.0 |  |
| Mammary bud, left |  |  |  |
| D_mean_ [95% CI] | 0.4 [0.1, 0.6] | 0.8 [0.6, 1.1] | <0.01 |
| min-max | 0.1 – 1.0 | 0.3 – 1.2 |  |
| Mammary bud, right |  |  |  |
| D_mean_ [95% CI] | 0.7 [-0.2, 1.6] | 2.3 [-0.9, 5.6] | 0.27 |
| min-max | 0.1 – 3.3 | 0.2 – 12.0 |  |
| **Abbreviations:** OARs, organs at risk; Gy, Gray; D_mean_, mean dose; 95% CI, 95% Confidence Interval; VMAT, Volumetric-Modulated Arc Therapy; AP/PA, Anterior-Posterior/Posterior-Anterior photon beam radiotherapy. | | | |
